# The benefit of combining curcumin, bromelain and harpagophytum to reduce inflammation in osteoarthritic synovial cells

**DOI:** 10.1186/s12906-021-03435-7

**Published:** 2021-10-14

**Authors:** Sybille Brochard, Julien Pontin, Benoit Bernay, Karim Boumediene, Thierry Conrozier, Catherine Baugé

**Affiliations:** 1 EA7451 BioConnect, Université de Caen Normandie, UNICAEN, 14032 Caen, France; 2 Proteogen platform, Normandie Univ, UNICAEN, Caen, France; 3 Rheumatology Department, Nord Franche-Comté Hospital, Trevenans, France

## Abstract

**Background:**

Osteoarthritis (OA) is the most common form of arthritis, affecting millions of people worldwide and characterised by joint pain and inflammation. It is a complex disease involving inflammatory factors and affecting the whole joint, including the synovial membrane. Since drug combination is widely used to treat chronic inflammatory diseases, a similar strategy of designing plant-derived natural products to reduce inflammation in OA joints may be of interest. In this study, we characterised the response of OA synovial cells to lipopolysaccharide (LPS) and investigated the biological action of the combination of curcumin, bromelain and harpagophytum in this original in vitro model of osteoarthritis.

**Methods:**

Firstly, human synovial cells from OA patients were stimulated with LPS and proteomic analysis was performed. Bioinformatics analyses were performed using Cytoscape App and SkeletalVis databases. Additionally, cells were treated with curcumin, bromelain and harpagophytum alone or with the three vegetal compounds together. The gene expression involved in inflammation, pain or catabolism was determined by RT-PCR. The release of the encoded proteins by these genes and of prostaglandin E2 (PGE2) were also assayed by ELISA.

**Results:**

Proteomic analysis demonstrated that LPS induces the expression of numerous proteins involved in the OA process in human OA synovial cells. In particular, it stimulates inflammation through the production of pro-inflammatory cytokines (Interleukin-6, IL-6), catabolism through an increase of metalloproteases (MMP-1, MMP-3, MMP-13), and the production of pain-mediating neurotrophins (Nerve Growth Factor, NGF). These increases were observed in terms of mRNA levels and protein release. LPS also increases the amount of PGE2, another inflammation and pain mediator. At the doses tested, vegetal extracts had little effect: only curcumin slightly counteracted the effects of LPS on NGF and MMP-13 mRNA, and PGE2, IL-6 and MMP-13 release. In contrast, the combination of curcumin with bromelain and harpagophytum reversed lots of effects of LPS in human OA synovial cells. It significantly reduced the gene expression and/or the release of proteins involved in catabolism (MMP-3 and -13), inflammation (IL-6) and pain (PGE2 and NGF).

**Conclusion:**

We have shown that the stimulation of human OA synovial cells with LPS can induce protein changes similar to inflamed OA synovial tissues. In addition, using this model, we demonstrated that the combination of three vegetal compounds, namely curcumin, bromelain and harpagophytum, have anti-inflammatory and anti-catabolic effects in synovial cells and may thus reduce OA progression and related pain.

**Supplementary Information:**

The online version contains supplementary material available at 10.1186/s12906-021-03435-7.

## Introduction

Osteoarthritis (OA) is a debilitating and painful disease characterised by inflammation of the synovial membrane and the progressive destruction of articular cartilage [1, 2]. It is one of the top 10 causes of physical disability [3]. However, its aetiology and pathogenesis are still not fully understood. Long considered a simple degenerative cartilage disease, OA is now described as a global joint disease [4]. To date, no treatment has been able to reverse OA progression.

Although OA is not classified as an inflammatory disease, many reports suggest that inflammation could be a major driver of OA development. In fact, elevated joint inflammation has been correlated with progression of the disease [5]. Therefore, although OA pathogenesis remains unclear, inflammation is widely regarded as an extremely important factor for the progression of this disease [2, 6–8] and pain severity [9–11].

Synovitis, i.e. the inflammation of synovial tissues, is common in OA [12] and is mediated, in part, by fibroblast-like synoviocytes (FLS). These cells play an important role in OA inflammation and joint destruction, primarily by secreting a wide range of proinflammatory mediators, such as IL-6 and prostaglandin E2 (PGE2) [12], which leads the release of neurotrophins such as NGF, leading to pain during OA. They also secrete various type of proteases, including matrix metalloproteinases (MMPs) and the A Disintegrin and Metalloproteinase with Thrombospondin Motifs family (or enzymes) (ADAMTS) [13], thus promoting the degradation of extracellular cell matrix (ECM) and further aggravating the progression of OA. Therefore, alleviating synovial inflammation may prevent the onset or minimise the progression of OA and symptoms [2, 14–16]. Conventional anti-inflammatory drugs are nonsteroidal anti-inflammatory drugs (NSAIDs) [17]. However, these entail several side effects and drug interactions, including the risk of gastrointestinal, cardiovascular and kidney problems. The use of natural compounds may be a relevant alternative.

Herbal medicine has been used since ancient times for healing purposes and is still used today. Curcumin (CUR), which is extracted from the rhizome of *Curcuma longa* L., is one of the most ancient medicinal herbs and is widely used in human health due to its various therapeutic features, such as anti-inflammatory, antioxidant, anticancer and antimicrobial effects [18]. In patients with OA, oral administration of curcumin improves the clinical manifestation of the disease [19–22], improves quality of life and enables a decrease in the consumption of NSAIDs [23]. This beneficial effect of curcumin is associated with its ability to reduce OA inflammation in cells, animal models and human studies [24, 25]. The action of curcumin may be reinforced by combining with other natural compounds [18, 26].

The purpose of the study was to investigate the effects of the combination of curcumin (CUR), bromelain (BRO) — a food obtained from pineapple which has analgesic properties [27] — and harpagophytum (HAR) — a traditional remedy for articular diseases [28] — on inflammation in an original in vitro model of osteoarthritis, using human synovial cells treated with lipopolysaccharide (LPS).

## Material and methods

### Reagents

Lipopolysaccharide (LPS) from *E.Coli* (Sigma Aldrich, Saint Louis, USA) was dissolved in phosphate buffer saline with no Calcium or Magnesium (DPBS, Lonza, Basel, Switzerland) in order to reach a concentration of 1 mg/ml, and was used once a final concentration of 1 μg/ml was attained. Curcumin (Turmeric extract granules, 95% curcuminoids, Natural, St Sylvain d’Anjou, France) was resuspended in dimethyl sulfoxide (DMSO, Dutscher, Bernolsheim, France). For the harpagophytum (*Harpagophytum procumbens*, Biosearch Life, Granada, Spain) and bromelain (Bromelain 2500 GDU, Cambridge Commodities Ltd., Ely, UK) extracts, the suspension was carried out in DPBS. The concentration of curcumin used was 13 μM (stock solution 130 mM), bromelain 14.7 μg/ml (stock solution 147 mg/ml) and harpagophytum 36 μg/ml (360 mg/ml).

### Culture cells and treatments

Human synoviocytes were recovered from the synovial membrane of six patients undergoing hip replacement surgery (mean age = 75 years). The cells were released by enzymatic digestion of the synovial membrane with collagenase type I (2 mg/ml, 12 h; ThermoFisher, Waltham, USA). The cells were cultured in Dulbecco’s Modified Eagle Medium high glucose with glutamine and sodium pyruvate (DMEM, Dutscher), supplemented with 10% Foetal Bovine Serum (FBS, Dutscher) and penicillin-streptomycin (Lonza), then incubated at 37 °C in a humid atmosphere containing 5% CO_2_.

To achieve the desired number of cells, passages were performed. The cells were rinsed with DPBS, then detached with 0.05% trypsin (ThermoFisher). The cells were recovered in a culture medium and seeded at approximately 7500 cells/cm^2^. The absence of mycoplasmas was checked by PCR.

The cells were processed at the confluence stage. Treatments were diluted in a new culture medium to the desired concentration. Each molecule was tested alone or in the presence of LPS. The three molecules were also tested together in order to see the effects of the combination of these three extracts, in the presence and absence of LPS.

### Protein extraction

Cells were lysed and protein extracted using Radio Immuno Precipitation Assay (RIPA) Buffer (50 mM Tris-HCl pH 7.5; 1% Igepal CA-630; 150 mM NaCl; 1 mM EGTA; 1 mM NaF; 0.25% Na-deoxycholate; Distilled water), and supplemented with a protease inhibitor (Leupeptin 1 mg/m; Phenyl methyl sulfonyl fluoride 200 m; pepstatin A 1 mg/ml) and a phosphatase inhibitor (sodium orthovanadate 200 mM) as previously described [29].

### Proteomic experiment

Five μg of each protein extract was prepared using a modified Gel-aided Sample Preparation protocol [30]. Samples were digested with trypsin/Lys-C overnight at 37 °C. For nano-LC fragmentation, protein or peptide samples were first desalted and concentrated onto a μC18 Omix (Agilent) before analysis.

The chromatography step was performed on a nanoElute (Bruker Daltonics) ultra-high pressure nano-flow chromatography system. Approximatively 200 ng of each peptide sample was concentrated onto a C18 PepMap 100 (5 mm × 300 μm i.d.) precolumn (Thermo Scientific) and separated at 50 °C onto a reversed phase ReproSil column (25 cm × 75 μm i.d.) packed with 1.6 μm C18 coated porous silica beads (IonOpticks). Mobile phases consisted of 0.1% formic acid, 99.9% water (v/v) (A) and 0.1% formic acid in 99.9% ACN (v/v) (B). The nanoflow rate was set at 400 nl/min, and the gradient profile was as follows: from 2 to 15% B within 60 min, followed by an increase to 25% B within 30 min and further to 37% within 10 min, followed by a washing step at 95% B and re-equilibration.

Mass spectrometry (MS) experiments were carried out on an TIMS-TOF pro mass spectrometer (Bruker Daltonics) with a modified nano electrospray ion source (CaptiveSpray, Bruker Daltonics). The system was calibrated each week and mass precision was better than 1 ppm. A 1600 spray voltage with a capillary temperature of 180 °C was typically employed for ionising. MS spectra were acquired in the positive mode in the mass range of 100 to 1700 m/z. In the experiments described here, the mass spectrometer was operated in Parallel Accumulation Serial Fragmentation (PASEF) mode with the exclusion of single charged peptides [31]. A number of 10 PASEF MS/MS scans were performed for 1.25 s from a charge range of 2–5.

Before the post-processing, the samples were analysed using Preview software (Protein Metrics) in order to estimate the quality of the tryptic digestion and predict the post-translational modifications present. The result, below, is used for the ‘bank research/identification’ part. The fragmentation pattern was used to determine the sequence of the peptide. Database searching was performed using the Peaks X+ software. A UniProt *Homo sapiens* database (October 2020) was used. The variable modifications allowed were as follows: N-terminal acetylation, methionine oxidation, Deamidation (NQ), Methylation (KR) and Carbamylation. In addition, C-Propionamide was set as the fixed modification. ‘Trypsin’ was selected as Specific. Mass accuracy was set to 30 ppm and 0.05 Da for the MS and MS/MS modes respectively. Data were filtered according to a false discovery rate (FDR) of 0.5% and protein redundancy was eliminated on the basis of proteins being evidenced by the same set or subset of peptides.

### Identification of differentially expressed proteins

To quantify the relative levels of protein abundance between different groups, samples were analysed using the label-free quantification feature of PEAKS X+ software [32]. Feature detection was separately performed on each sample by the expectation-maximisation algorithm. The features of the same peptide from all replicates of each sample were aligned through the retention time alignment algorithms. Mass error tolerance was set at 30 ppm, Ion Mobility Tolerance (1/k0) at 0.07 and retention time tolerance at 10 min. Normalisation factors of the samples were obtained by the total ion current (TIC) of each sample. Quantification of the protein abundance level was calculated using the sum area of the top three unique peptides. A 1.5-fold increase in relative abundance and a significance of ≥5 using ANOVA as the significance method were used to determine those enriched proteins.

### Enrichment analysis and comparison with datasets related to skeletal biology

The heatmap technique was performed with a Spearman clustering method using the ComplexHeatmap R package.

Enrichments in the molecular processes, cellular processes and pathways (KEGG and Reactome) were performed using the ClueGo App from the Cytoscape software. Network specificity was set to medium; the GO tree interval was set between 2 and 4. Clusters were performed using a selection set to a minimum of three genes in addition to 4% of genes. Enrichments were performed using a Bonferroni step-down method.

Additionally, differentially expressed proteins were compared to existing gene expression datasets related to skeletal biology using the SkeletalVis application (http://skeletalvis.ncl.ac.uk/skeletal/, [33]). Proteins encoded by genes associated with osteoarthritis joint damage in animals were also identified using OATargets databases [34].

### RNA extraction and RT-PCR

RNA was extracted from the cell layer using the RNeasy mini kit (Qiagen, Hilden, Germany) in accordance with the supplier’s protocol. Then, DNase treatment and reverse transcription were carried out using the DNase I kit (Sigma Aldrich) and the reverse transcriptase M-MLV (Invitrogen, Carlsbad, USA) as previously described [35]. Next, cDNA was amplified by real-time PCR using a PCR master Mix (Power SYBR Green, Applied biosystems, Courtaboeuf, France) and read on the Step One Plus Real Time PCR system (Applied Biosystems) with the following primers: RPL13A Forward: 5′-GAGGTATGCTGCCCCACAAA-3′ and Reversed: 5′-GTGGGATGCCGTCAAACAC-3′; NGF Forward: 5′-AGCGCAGCGAGTTTTGG-3′ and Reversed: 5′-AGAAAGCTGCTCCCTTGGTA-3′; IL-6 Forward: 5′-CACACAGACAGCCACTCACC-3′ and Reversed: 5′-TTTCACCAGGCAAGTCTCCT-3′; MMP-1 Forward: 5′-GAAGCTGCTTACGAATTTGCCG-3′ and Reversed: 5′-CCAAAGGAGCTGTAGATGTCCT-3′; MMP-3 Forward: 5′-TAAAGACAGGCACTT TTGGCGC-3′ and Reversed: 5′-TTGGGTATCCAGCTCGTACCTC-3′; MMP-13 Forward: 5′-AAGGAGCATGGCGACTTCT-3′ and Reversed: 5′-TGGCCCAGGAGGAAAAGC-3′. The relative mRNA level was calculated using the 2^−ΔΔCT^ method. RPL13a was used as the invariant housekeeping gene. The decision to opt for this gene was based on our previous experience in the field [29, 35, 36].

### Elisa

PGE2 and MMP released into conditioned media were quantified using a commercially available enzyme immunoassay kit (R&D Biosystem) as previously described [29]. For IL-6, we proceeded in the same way but using the Human beta-NGF ELISA Kit and the Human IL-6 ELISA kit (Sigma Aldrich). The immunoassays were all carried out in accordance with the manufacturer’s protocol. Absorbance was determined at 450 nm with a wavelength correction set at 540 nm using the Multiskan GO spectrophotometer (Thermo Scientific).

### Statistical analyses

All results are expressed as the mean value of three or four patients (biological replicates) + the standard error of the mean (SEM). Statistical analyses were carried out on the GraphPad prism 8 software. After checking the normal distribution of samples, two-way ANOVA tests were used for multiple comparisons. In significant cases, Tukey’s multiple comparisons test for matched samples was performed as a post-hoc analysis. *P*-values < 0.05 were considered significant.

## Results

### Stimulation of human OA synovial cells with LPS, an efficient OA model in vitro

**Fig. 1:**
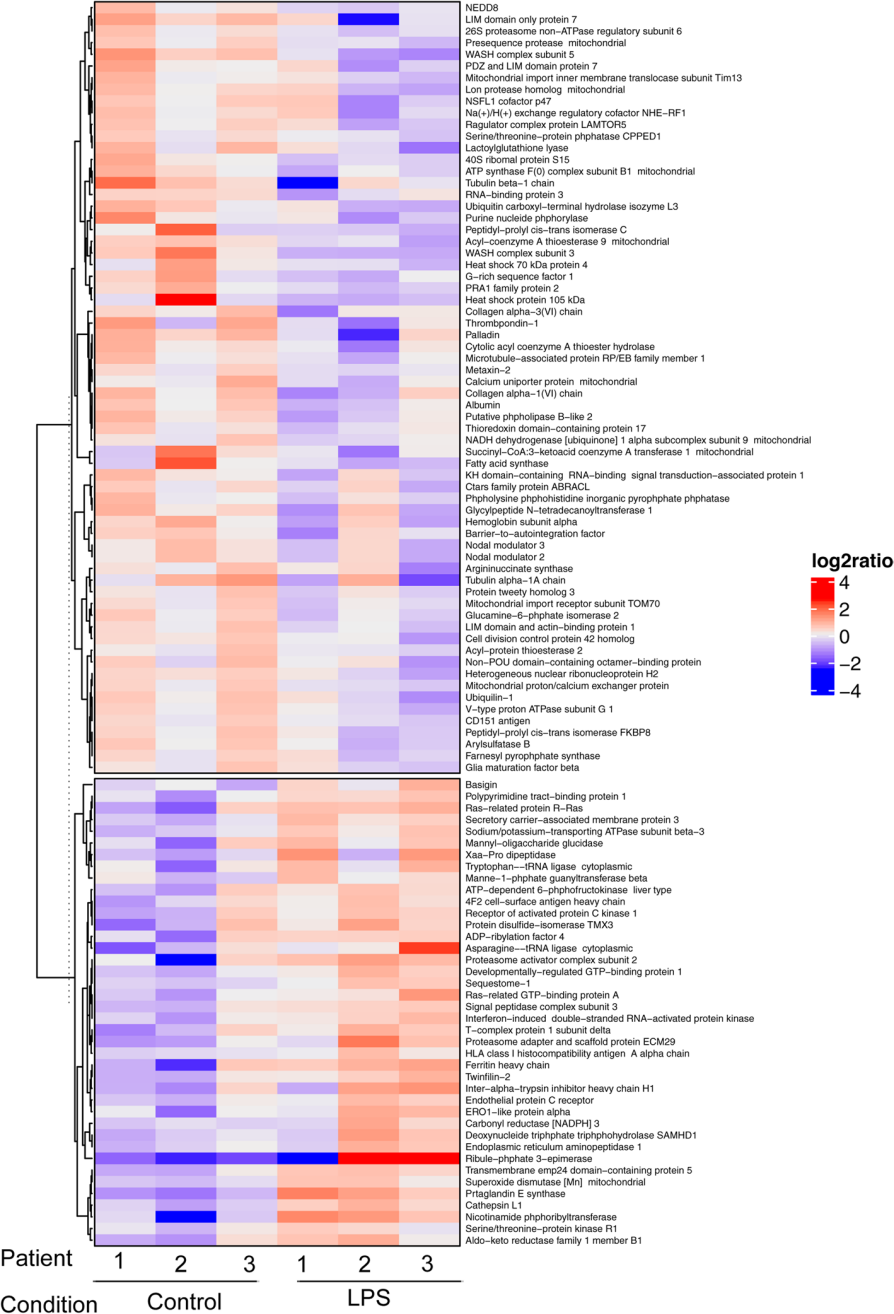
Heatmap showing differentially expressed proteins by LPS in human synovial cells. Human synovial cells from three different patients were treated with LPS (1 μg/ml) for 48 h. At the end of the experiments, proteins were extracted and proteomic analysis was performed. Differentially expressed proteins from among the control group and the LPS group is shown (*n* = 3)

**Fig. 2:**
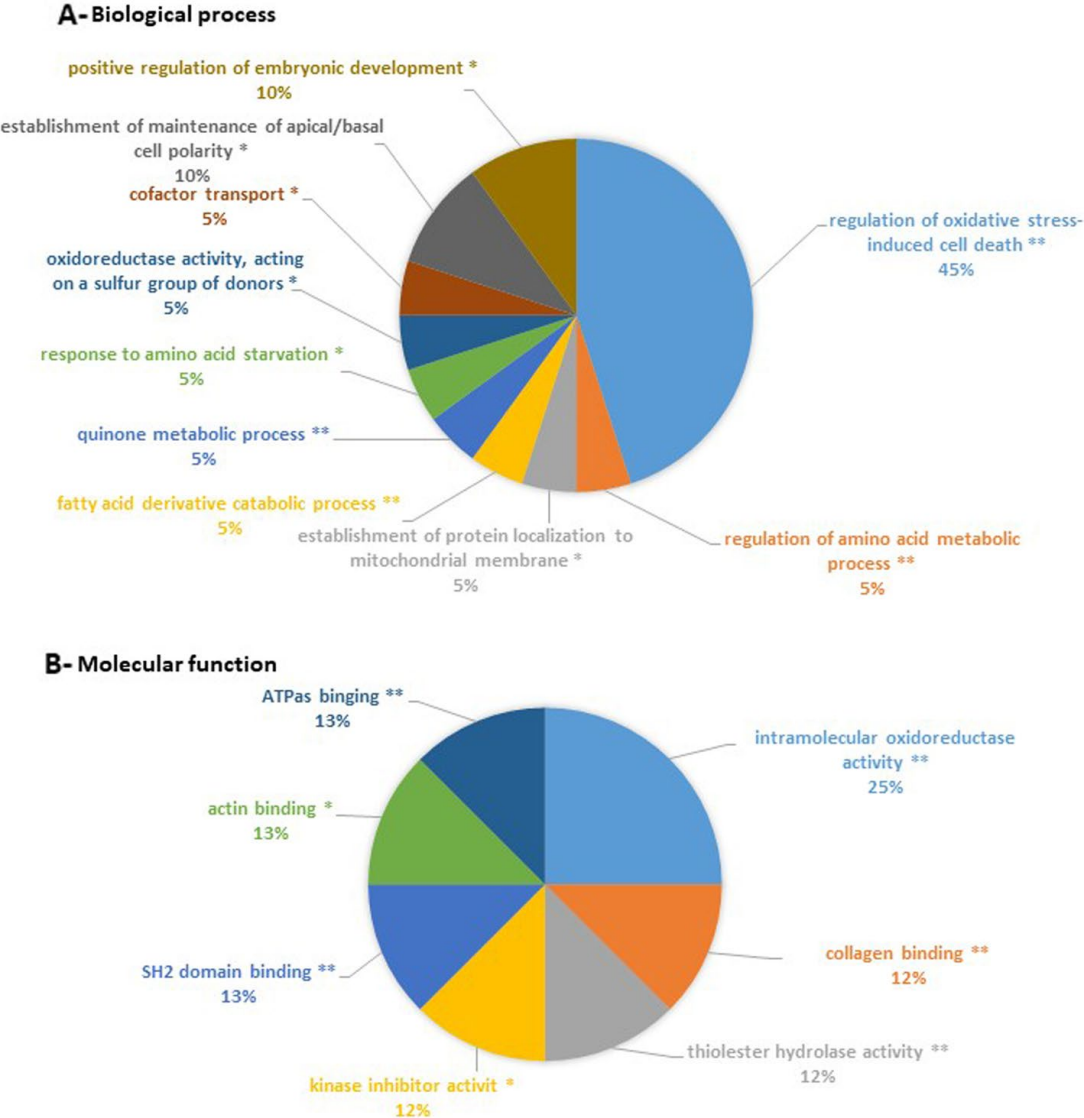
Enrichment in the biological process and molecular function. From differentially expressed proteins between the control group and the LPS group (Fig. 1), enrichments in a biological process (**A**) and molecular process (**B**) were performed. The diagram shows part of each GO Term, which were statistically enriched. *: *p*-value< 0.05, **: *p*-value< 0.01

**Fig. 3:**
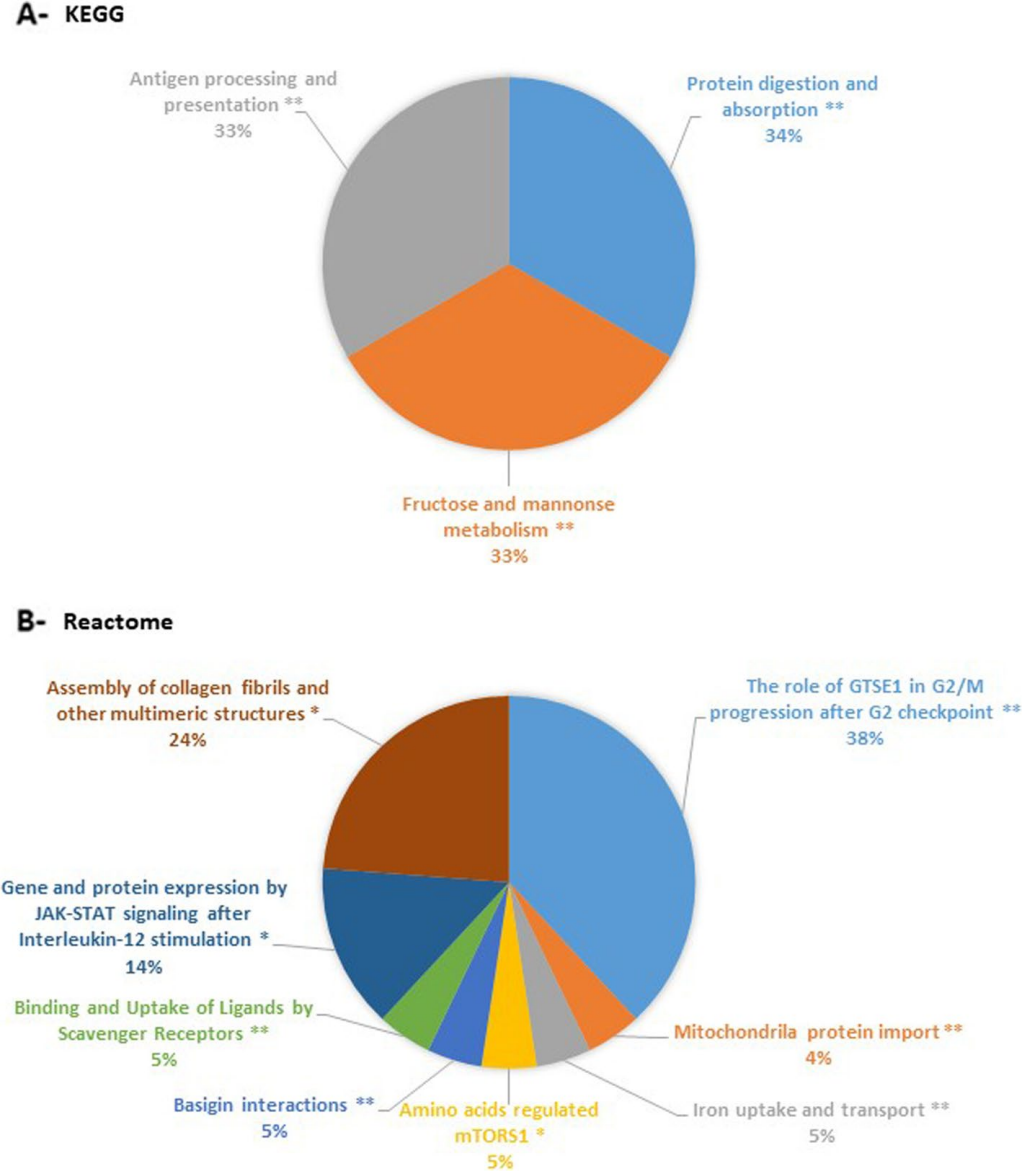
Enrichment in functional pathways. From differentially expressed proteins between the control group and the LPS group (Fig. 1), enrichments pathways using KEGG (**A**) or Reactome (**B**) datasets were performed. The diagram shows part of each GO Term, which were statistically enriched. *: *p*-value< 0.05, **: *p*-value< 0.01

Lipopolysaccharide (LPS) has recently been considered a stimulus which is able to trigger inflammation and OA onset, and is used to model the inflammatory component of OA. Therefore, we planned to test the effects of curcumin, harpagophytum and bromelain in human OA synovial cells stimulated with LPS. Before doing so, we wanted to validate the model and its ability to model OA inflammation. So, we conducted a proteomic analysis to define the differentially expressed proteins from among unstimulated OA synovial cells and LPS-stimulated OA synovial cells. Two thousand nine hundred seventeen proteins were identified in the control group, and 3011 in the LPS treated-group. Among them, 106 proteins were differentially expressed between the two groups (Peaks Sign > 5, Fold-change > 1.5, Fig. 1 and Table 1). More precisely, 66 proteins (i.e. 62%) were significantly downregulated by LPS, and 40 (i.e. 38%) were upregulated by LPS. ClueGo analysis revealed that these differentially expressed proteins are mainly involved in the biological processes of oxidative stress-induced cell death (45%, *p*-value < 0.01) and in the molecular processes of intramolecular oxidoreductase activity (25%, *p*-value < 0.01) and collagen binding (12.5%, *p*-value < 0.01) (Fig. 2, Tables 2 and 3). Furthermore, KEGG pathway enrichment (Fig. 3A, Table 4) showed the presence of proteins involved in protein digestion and absorption, fructose and mannose metabolism, and antigen processing and presentation (33% for each, *p*-value < 0.01). Enrichment using Reactome (Fig. 3B, Table 5) also showed the presence of proteins involved in the assembly of collagen fibrils and other multimeric structures (24%, *p*-value < 0.05).

**Table 1:** List of deregulated proteins in LPS-stimulated synovial cells

Accession	Group Profile (Ratio)	Gene names (primary)	Description	OA associated	human OA DEG	induced OA DEG	OA gene interaction	skeletal phenotype
***Proteins down-regulated by LPS***								
Q92598	0.23	HSPH1	Heat shock protein 105 kDa	false	2	8	14	false
P49327	0.25	FASN	Fatty acid synthase	false	3	7	17	false
Q9Y3C0	0.26	WASHC3	WASH complex subunit 3	false	1	1	3	false
Q9H4B7	0.32	TUBB1	Tubulin beta-1 chain	false	5	1	3	false
P45877	0.36	PPIC	Peptidyl-prolyl cis-trans isomerase C	false	8	9	0	false
Q12768	0.43	WASHC5	WASH complex subunit 5	false	0	0	2	false
P07996	0.45	THBS1	Thrombpondin-1	true	4	2	19	true
Q8WWI1	0.45	LMO7	LIM domain only protein 7	false	3	5	7	false
Q8WX93	0.47	PALLD	Palladin	false	3	2	5	false
Q8NHP8	0.48	PLBD2	Putative phpholipase B-like 2	false	0	0	2	false
Q8NE86	0.49	MCU	Calcium uniporter protein mitochondrial	false	0	0	0	false
O60831	0.51	PRAF2	PRA1 family protein 2	false	1	3	0	false
Q9UMX0	0.52	UBQLN1	Ubiquilin-1	false	1	1	15	false
Q9NR12	0.52	PDLIM7	PDZ and LIM domain protein 7	false	5	3	7	false
O00154	0.52	ACOT7	Cytolic acyl coenzyme A thioester hydrolase	false	0	3	1	false
Q9Y305	0.53	ACOT9	Acyl-coenzyme A thioesterase 9 mitochondrial	false	0	4	6	false
Q71U36	0.53	TUBA1A	Tubulin alpha-1A chain	false	1	1	31	false
P15374	0.53	UCHL3	Ubiquitin carboxyl-terminal hydrolase isozyme L3	false	0	7	5	false
Q04760	0.53	GLO1	Lactoylglutathione lyase	false	0	3	3	true
P30419	0.53	NMT1	Glycylpeptide N-tetradecanoyltransferase 1	false	1	1	4	false
P55809	0.54	OXCT1	Succinyl-CoA:3-ketoacid coenzyme A transferase 1 mitochondrial	false	2	4	3	false
O43504	0.55	LAMTOR5	Ragulator complex protein LAMTOR5	false	0	1	4	false
P62841	0.55	RPS15	40S ribomal protein S15	false	1	5	9	false
P36776	0.55	LONP1	Lon protease homolog mitochondrial	false	4	3	7	false
Q12849	0.55	GRSF1	G-rich sequence factor 1	false	0	1	2	false
Q5JRX3	0.55	PITRM1	Presequence protease mitochondrial	false	2	3	2	false
Q8TDQ7	0.55	GNPDA2	Glucamine-6-phphate isomerase 2	false	0	1	1	false
P34932	0.56	HSPA4	Heat shock 70 kDa protein 4	false	0	2	39	false
Q15691	0.56	MAPRE1	Microtubule-associated protein RP/EB family member 1	false	0	0	13	false
P24539	0.56	ATP5PB	ATP synthase F(0) complex subunit B1 mitochondrial	false	0	0	6	false
P00491	0.56	PNP	Purine nucleide phphorylase	false	6	3	1	false
P69905	0.56	HBA1; HBA2	Hemoglobin subunit alpha	false	7	0	3	false
Q15008	0.57	PSMD6	26S proteasome non-ATPase regulatory subunit 6	false	0	5	4	false
P02768	0.57	ALB	Albumin	false	2	0	9	false
Q9UHB6	0.57	LIMA1	LIM domain and actin-binding protein 1	false	2	4	9	true
Q15843	0.57	NEDD8	NEDD8	false	0	3	9	false
P15848	0.58	ARSB	Arylsulfatase B	false	3	6	1	true
O95202	0.58	LETM1	Mitochondrial proton/calcium exchanger protein	false	0	2	3	false
P12109	0.59	COL6A1	Collagen alpha-1(VI) chain	true	6	12	9	false
Q9UNZ2	0.59	NSFL1C	NSFL1 cofactor p47	false	0	1	8	false
Q9Y5L4	0.59	TIMM13	Mitochondrial import inner membrane translocase subunit Tim13	false	0	3	3	false
P55795	0.61	HNRNPH2	Heterogeneous nuclear ribonucleoprotein H2	false	0	2	5	true
Q9H008	0.61	LHPP	Phpholysine phphohistidine inorganic pyrophphate phphatase	false	4	5	0	false
P12111	0.62	COL6A3	Collagen alpha-3(VI) chain	false	6	16	2	true
O14745	0.62	SLC9A3R1	Na(+)/H(+) exchange regulatory cofactor NHE-RF1	false	1	4	11	true
P98179	0.62	RBM3	RNA-binding protein 3	false	1	1	5	false
Q14318	0.62	FKBP8	Peptidyl-prolyl cis-trans isomerase FKBP8	false	1	2	10	true
O94826	0.62	TOMM70	Mitochondrial import receptor subunit TOM70	false	0	2	4	false
P48509	0.62	CD151	CD151 antigen	false	0	1	0	false
O75348	0.62	ATP6V1G1	V-type proton ATPase subunit G 1	false	1	2	0	false
P60953	0.63	CDC42	Cell division control protein 42 homolog	true	0	0	19	true
Q9BRA2	0.63	TXNDC17	Thioredoxin domain-containing protein 17	false	1	1	0	false
Q9BRF8	0.63	CPPED1	Serine/threonine-protein phphatase CPPED1	false	2	0	0	false
Q07666	0.64	KHDRBS1	KH domain-containing RNA-binding signal transduction-associated protein 1	false	0	2	16	true
Q16795	0.64	NDUFA9	NADH dehydrogenase [ubiquinone] 1 alpha subcomplex subunit 9 mitochondrial	false	0	2	6	false
O75431	0.64	MTX2	Metaxin-2	false	0	1	0	false
Q9C0H2	0.64	TTYH3	Protein tweety homolog 3	false	1	4	0	false
Q5JPE7	0.64	NOMO2	Nodal modulator 2	false	0	0	1	false
P69849	0.64	NOMO3	Nodal modulator 3	false	0	0	1	false
P00966	0.65	ASS1	Argininuccinate synthase	false	4	2	4	false
O75531	0.65	BANF1	Barrier-to-autointegration factor	false	0	2	2	false
O95372	0.65	LYPLA2	Acyl-protein thioesterase 2	false	0	0	1	false
Q9P1F3	0.65	ABRACL	Ctars family protein ABRACL	false	3	2	0	false
Q15233	0.66	NONO	Non-POU domain-containing octamer-binding protein	false	0	2	16	false
P14324	0.66	FDPS	Farnesyl pyrophphate synthase	false	0	2	3	false
P60983	0.66	GMFB	Glia maturation factor beta	false	1	2	1	false
***Protein up-regulated by LPS***								
Q96AT9	51.49	RPE	Ribulose-phosphate 3-epimerase	false	0	0	3	false
O14684	4.09	PTGES	Prostaglandin E synthase	false	8	4	0	false
P43490	3.35	NAMPT	Nicotinamide phphoribyltransferase	true	8	0	4	false
O43776	2.66	NARS1	Asparagine--tRNA ligase cytoplasmic	false	0	0	3	false
Q5VYK3	2.56	ECPAS	Proteasome adapter and scaffold protein ECM29	false	1	0	8	false
P12955	2.47	PEPD	Xaa-Pro dipeptidase	false	0	5	5	true
P10301	2.33	RRAS	Ras-related protein R-Ras	false	3	1	5	false
P19827	2.27	ITIH1	Inter-alpha-trypsin inhibitor heavy chain H1	false	0	1	1	false
P15121	2.04	AKR1B1	Aldo-keto reductase family 1 member B1	false	0	4	3	false
O14828	1.96	SCAMP3	Secretory carrier-associated membrane protein 3	false	1	1	4	false
P07711	1.95	CTSL	Cathepsin L1	false	4	0	3	false
Q6IBS0	1.92	TWF2	Twinfilin-2	false	0	5	0	false
P04179	1.9	SOD2	Superoxide dismutase [Mn] mitochondrial	true	7	7	8	false
P54709	1.85	ATP1B3	Sodium/potassium-transporting ATPase subunit beta-3	false	1	3	3	false
Q9Y3Z3	1.84	SAMHD1	Deoxynucleide triphphate triphphohydrolase SAMHD1	false	1	2	3	false
Q9Y3A6	1.84	TMED5	Transmembrane emp24 domain-containing protein 5	false	3	3	0	false
Q13501	1.79	SQSTM1	Sequestome-1	false	3	1	42	true
Q96JJ7	1.79	TMX3	Protein disulfide-isomerase TMX3	false	0	0	0	false
O75828	1.78	CBR3	Carbonyl reductase [NADPH] 3	false	2	5	3	false
P35613	1.78	BSG	Basigin	false	2	0	7	false
P26599	1.76	PTBP1	Polypyrimidine tract-binding protein 1	false	2	0	11	false
Q9Y295	1.76	DRG1	Developmentally-regulated GTP-binding protein 1	false	0	1	2	false
Q7L523	1.74	RRAGA	Ras-related GTP-binding protein A	false	0	3	2	false
P61009	1.7	SPCS3	Signal peptidase complex subunit 3	false	2	1	1	false
Q96HE7	1.69	ERO1A	ERO1-like protein alpha	false	5	1	1	false
Q9UL46	1.66	PSME2	Proteasome activator complex subunit 2	false	0	0	2	false
Q13724	1.63	MOGS	Mannyl-oligaccharide glucidase	false	0	2	5	false
Q9Y5P6	1.63	GMPPB	Manne-1-phphate guanyltransferase beta	false	2	2	1	false
P19525	1.61	EIF2AK2	Interferon-induced double-stranded RNA-activated protein kinase	false	1	1	18	false
P02794	1.59	FTH1	Ferritin heavy chain	false	3	1	5	false
P18085	1.58	ARF4	ADP-ribylation factor 4	false	2	10	10	false
P23381	1.57	WARS1	Tryptophan--tRNA ligase cytoplasmic	false	0	0	3	false
P63244	1.56	RACK1	Receptor of activated protein C kinase 1	false	1	0	22	false
Q9NZ08	1.56	ERAP1	Endoplasmic reticulum aminopeptidase 1	false	1	1	3	false
O95747	1.55	OXSR1	Serine/threonine-protein kinase R1	false	1	1	6	false
P17858	1.54	PFKL	ATP-dependent 6-phphofructokinase liver type	false	1	3	6	false
P08195	1.53	SLC3A2	4F2 cell-surface antigen heavy chain	false	6	0	7	false
Q9UNN8	1.53	PROCR	Endothelial protein C receptor	false	4	8	0	false
P04439	1.51	HLA-A	HLA class I histocompatibility antigen A alpha chain	false	1	0	5	false
P50991	1.5	CCT4	T-complex protein 1 subunit delta	false	0	3	11	false

**Table 2:** Enrichment in biological processes

GOID	GOTerm	Term PValue	% Associated Genes	Nr. Genes	Associated Genes Found
GO:0006521	regulation of cellular amino acid metabolic process	0.007	4.48	3	[BSG, PSMD6, PSME2]
GO:0016667	oxidoreductase activity, acting on a sulfur group of donors	0.005	5.00	3	[ERO1A, TMX3, TXNDC17]
GO:0071230	cellular response to amino acid stimulus	0.001	5.56	4	[ASS1, COL6A1, LAMTOR5, RRAGA]
GO:0072523	purine-containing compound catabolic process	0.004	5.36	3	[ACOT7, PNP, SAMHD1]
GO:1901569	fatty acid derivative catabolic process	0.000	18.75	3	[ACOT7, LYPLA2, OXCT1]
GO:1901661	quinone metabolic process	0.001	8.11	3	[AKR1B1, CBR3, NDUFA9]
GO:1990928	response to amino acid starvation	0.003	5.88	3	[EIF2AK2, FASN, RRAGA]
GO:0034198	cellular response to amino acid starvation	0.003	6.25	3	[EIF2AK2, FASN, RRAGA]
GO:0070671	response to interleukin-12	0.005	5.08	3	[CDC42, PSME2, SOD2]
GO:0035722	interleukin-12-mediated signaling pathway	0.004	5.36	3	[CDC42, PSME2, SOD2]
GO:0040019	positive regulation of embryonic development	0.002	6.98	3	[AKR1B1, OXSR1, RACK1]
GO:0071470	cellular response to osmotic stress	0.004	5.66	3	[AKR1B1, LETM1, OXSR1]
GO:0051181	cofactor transport	0.005	5.17	3	[BSG, OXSR1, SLC9A3R1]
GO:0072337	modified amino acid transport	0.001	10.00	3	[BSG, OXSR1, SLC9A3R1]
GO:0061245	establishment or maintenance of bipolar cell polarity	0.004	5.66	3	[ARF4, CDC42, SLC9A3R1]
GO:0035088	establishment or maintenance of apical/basal cell polarity	0.004	5.66	3	[ARF4, CDC42, SLC9A3R1]
GO:0045197	establishment or maintenance of epithelial cell apical/basal polarity	0.003	6.25	3	[ARF4, CDC42, SLC9A3R1]
GO:0007006	mitochondrial membrane organization	0.000	4.05	6	[ATP5PB, HSPA4, LETM1, MTX2, NMT1, TIMM13]
GO:0051205	protein insertion into membrane	0.007	4.48	3	[HSPA4, NMT1, TIMM13]
GO:0090151	establishment of protein localization to mitochondrial membrane	0.003	5.88	3	[HSPA4, NMT1, TIMM13]
GO:0051204	protein insertion into mitochondrial membrane	0.003	6.38	3	[HSPA4, NMT1, TIMM13]
GO:1902882	regulation of response to oxidative stress	0.000	4.90	5	[BSG, NONO, RACK1, SOD2, UBQLN1]
GO:1902883	negative regulation of response to oxidative stress	0.000	6.67	4	[BSG, NONO, RACK1, SOD2]
GO:0036473	cell death in response to oxidative stress	0.000	5.00	5	[BSG, NONO, RACK1, SOD2, UBQLN1]
GO:1900407	regulation of cellular response to oxidative stress	0.000	5.38	5	[BSG, NONO, RACK1, SOD2, UBQLN1]
GO:1900408	negative regulation of cellular response to oxidative stress	0.000	6.90	4	[BSG, NONO, RACK1, SOD2]
GO:0008631	intrinsic apoptotic signaling pathway in response to oxidative stress	0.003	6.25	3	[NONO, SOD2, UBQLN1]
GO:1903201	regulation of oxidative stress-induced cell death	0.000	6.33	5	[BSG, NONO, RACK1, SOD2, UBQLN1]
GO:0036475	neuron death in response to oxidative stress	0.001	9.09	3	[BSG, NONO, RACK1]
GO:1903202	negative regulation of oxidative stress-induced cell death	0.000	6.90	4	[BSG, NONO, RACK1, SOD2]
GO:1903203	regulation of oxidative stress-induced neuron death	0.001	10.00	3	[BSG, NONO, RACK1]
GO:1902175	regulation of oxidative stress-induced intrinsic apoptotic signaling pathway	0.001	9.68	3	[NONO, SOD2, UBQLN1]
GO:1903204	negative regulation of oxidative stress-induced neuron death	0.000	13.64	3	[BSG, NONO, RACK1]

**Table 3:** Enrichment in molecular functions

GOID	GOTerm	Term PValue	% Associated Genes	Nr. Genes	Associated Genes Found
GO:0005518	collagen binding	0.009	4.05	3	[COL6A1, CTSL, THBS1]
GO:0016790	thiolester hydrolase activity	0.000	10.00	4	[ACOT7, ACOT9, FASN, LYPLA2]
GO:0019210	kinase inhibitor activity	0.008	4.29	3	[GMFB, RACK1, WARS1]
GO:0042169	SH2 domain binding	0.002	6.52	3	[KHDRBS1, RACK1, SQSTM1]
GO:0042805	actinin binding	0.002	6.98	3	[LMO7, PALLD, PDLIM7]
GO:0051117	ATPase binding	0.002	4.30	4	[AKR1B1, ATP1B3, ATP6V1G1, NSFL1C]
GO:0016667	oxidoreductase activity, acting on a sulfur group of donors	0.005	5.00	3	[ERO1A, TMX3, TXNDC17]
GO:0016860	intramolecular oxidoreductase activity	0.000	6.67	4	[ERO1A, GNPDA2, PTGES, TMX3]

**Table 4:** Enrichment using KEGG

GOID	GOTerm	Term PValue	% Associated Genes	Nr. Genes	Associated Genes Found
KEGG:00051	Fructose and mannose metabolism	0.002	9.09	3	[AKR1B1, GMPPB, PFKL]
KEGG:04612	Antigen processing and presentation	0.003	5.13	4	[CTSL, HLA-A, HSPA4, PSME2]
KEGG:04974	Protein digestion and absorption	0.005	4.21	4	[ATP1B3, COL6A1, COL6A3, SLC3A2]

**Table 5:** Enrichment using reactome

GOID	GOTerm	Term PValue	% Associated Genes	Nr. Genes	Associated Genes Found
R-HSA:1268020	Mitochondrial protein import	0.001	6.25	4	[MTX2, PITRM1, TIMM13, TOMM70]
R-HSA:917937	Iron uptake and transport	0.009	5.17	3	[ATP6V1G1, FTH1, NEDD8]
R-HSA:9639288	Amino acids regulate mTORC1	0.008	5.45	3	[ATP6V1G1, LAMTOR5, RRAGA]
R-HSA:210991	Basigin interactions	0.001	12.00	3	[ATP1B3, BSG, SLC3A2]
R-HSA:2173782	Binding and Uptake of Ligands by Scavenger Receptors	0.000	9.52	4	[ALB, FTH1, HBA1, HSPH1]
R-HSA:447115	Interleukin-12 family signaling	0.009	5.26	3	[CDC42, PSME2, SOD2]
R-HSA:8950505	Gene and protein expression by JAK-STAT signaling after Interleukin-12 stimulation	0.003	7.89	3	[CDC42, PSME2, SOD2]
R-HSA:9020591	Interleukin-12 signaling	0.005	6.38	3	[CDC42, PSME2, SOD2]
R-HSA:1442490	Collagen degradation	0.012	4.69	3	[COL6A1, COL6A3, CTSL]
R-HSA:1474290	Collagen formation	0.005	4.44	4	[CD151, COL6A1, COL6A3, CTSL]
R-HSA:186797	Signaling by PDGF	0.009	5.17	3	[COL6A1, COL6A3, THBS1]
R-HSA:2022090	Assembly of collagen fibrils and other multimeric structures	0.001	6.56	4	[CD151, COL6A1, COL6A3, CTSL]
R-HSA:216083	Integrin cell surface interactions	0.004	4.71	4	[BSG, COL6A1, COL6A3, THBS1]
R-HSA:1632852	Macroautophagy	0.001	4.41	6	[LAMTOR5, RRAGA, SQSTM1, TOMM70, TUBA1A, TUBB1]
R-HSA:2995410	Nuclear Envelope (NE) Reassembly	0.019	4.00	3	[BANF1, TUBA1A, TUBB1]
R-HSA:389957	Prefoldin mediated transfer of substrate to CCT/TriC	0.001	10.71	3	[CCT4, TUBA1A, TUBB1]
R-HSA:389958	Cooperation of Prefoldin and TriC/CCT in actin and tubulin folding	0.002	9.38	3	[CCT4, TUBA1A, TUBB1]
R-HSA:389960	Formation of tubulin folding intermediates by CCT/TriC	0.001	12.00	3	[CCT4, TUBA1A, TUBB1]
R-HSA:5626467	RHO GTPases activate IQGAPs	0.002	9.38	3	[CDC42, TUBA1A, TUBB1]
R-HSA:8852276	The role of GTSE1 in G2/M progression after G2 checkpoint	0.000	6.49	5	[MAPRE1, PSMD6, PSME2, TUBA1A, TUBB1]
R-HSA:9663891	Selective autophagy	0.003	4.94	4	[SQSTM1, TOMM70, TUBA1A, TUBB1]

The signature comparison of the proteomic profiles of the control group and the LPS-stimulated synovial cells using the SkeletalVis database, which allowed us to explore skeletal biology-related expression datasets [33], suggested that deregulated proteins were encoded by genes which are also differentially expressed in several other OA models (suppl. Data 1), namely ‘Synovial cells from inflammatory and normal areas of osteoarthritis synovial membrane’ (signed Jaccard index (sig) = 0.015; z score = 5.08) and observed in ‘Rat model of surgically induced knee osteoarthritis’ (signed Jaccard (sig) = 0.0118; z score = 3.98). In addition, using OATargets databases [34], we were able to observe that several identified proteins were encoded by genes associated with OA, such as Thrombpondin-1 (THBS1), collagen alpha-1(VI) chain (COL6A1), superoxide dismutase [Mn] mitochondrial (SOD2) and Nicotinamide phosphoribosyltransferase (NAMPT) (Table 1). In addition, about half of these genes were also found at least once as a human OA DEG, and around 90% are known to interact with OA genes (Table 1).

Altogether, this proteomic analysis clearly confirms that LPS-stimulated synovial cells from OA human patients are a good model for studying the osteoarthritis process in vitro.

### LPS increases the expression of genes associated with inflammation, catabolism and pain

**Fig. 4:**
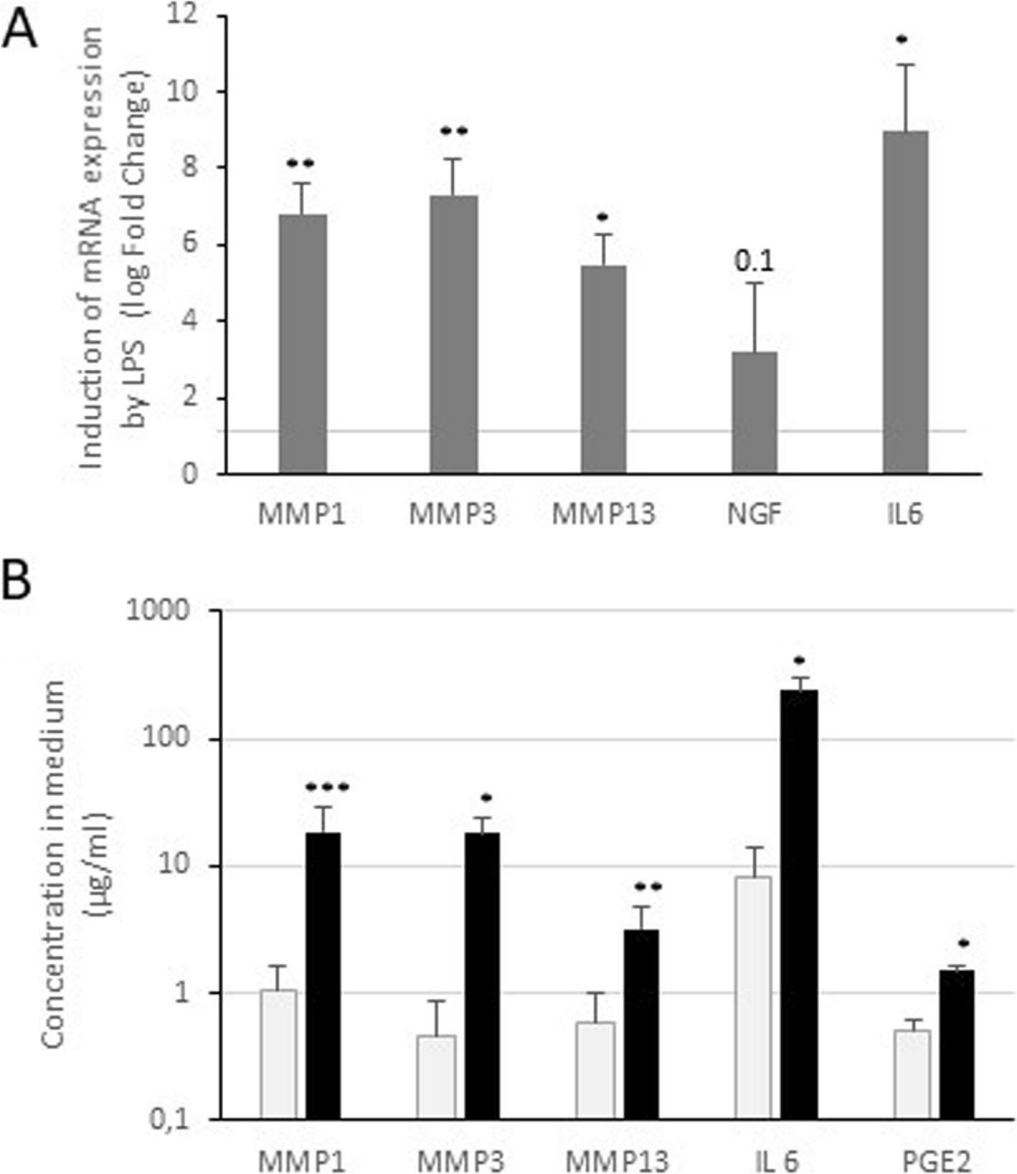
LPS induces gene expression and release in medium of catabolism, inflammation and pain markers. Human synovial cells were treated with LPS (1 μg/ml) for 24 h. **A** - At the end of the experiments, RNA was extracted. Relative mRNA expression of MMP-1, MMP-3, MMP-13, NGF and IL-6 was determined by RT-PCR. Values are compared to untreated cells and presented as a log Fold Change (compared to the control group). **B** - Culture media were collected and ELISA was performed to assayed MMP, IL-6 and PGE2 concentration in medium. Values are expressed as μg/ml medium (*n* = 4). *: *p*-value< 0.05, **: *p*-value< 0.01, ***: *p*-value< 0.001

Next, using a commonly targeted strategy, we investigated the effect of LPS treatment in human OA synovial cells. After 24 h of treatment, LPS stimulated inflammation through the production of pro-inflammatory cytokines (Interleukin-6, IL-6), catabolism through an increase of metalloproteases (MMP-1, MMP-3, MMP-13), and the production of pain-mediating neurotrophin (Nerve Growth Factor, NGF). These increases were observed in terms of mRNA levels and protein release. LPS also increased the amount of PGE2, another pain mediator (Fig. 4).

### The combination of curcumin with bromelain and harpagophytum significantly reduced the LPS-induced expression of genes associated with catabolism

**Fig. 5:**
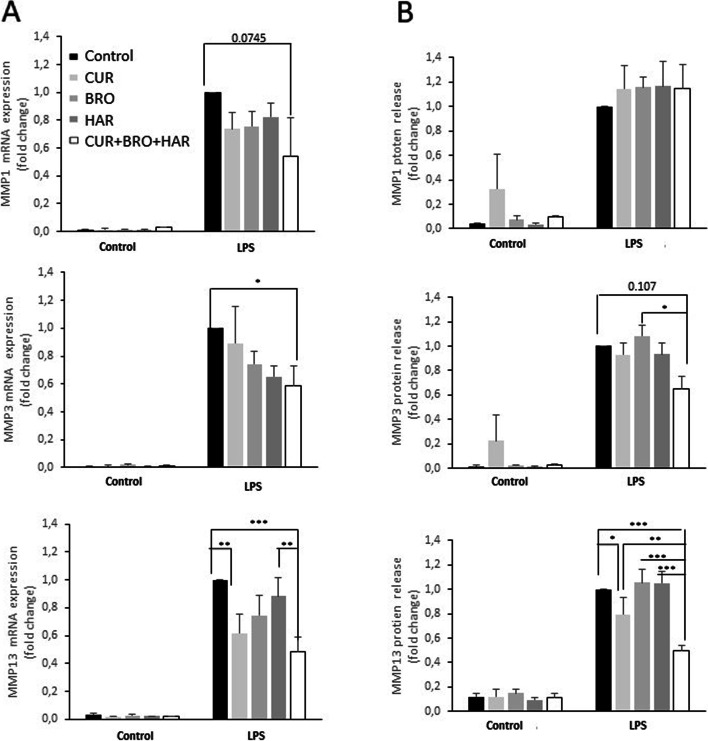
The combination of curcumin with bromelain and harpagophytum significantly reduced the LPS-induced expression of genes associated with catabolism. Human synovial cells were treated with LPS (1 μg/ml) for 24 h in the presence of curcumin (CUR, 13 μM), bromelain (BRO, 14.7 μg/ml) and harpagophytum (HAR, 36 μg/ml), and all three together. **A** - At the end of the experiments, RNA was extracted and media collected. Relative mRNA expression of MMP-1, MMP-3 and MMP-13 was determined by RT-PCR. **B** - Culture media were also collected and ELISA performed to assayed MMP release in medium. Values were compared to LPS-treated cells and presented as relative expression (compared to the LPS group). *n* = 3. *: *p*-value< 0.05, **: *p*-value< 0.01, ***: *p*-value< 0.001

Having validated our model, we continued by studying the effect of vegetal extracts (curcumin bromelain and harpagophytum) on OA-associated genes. On the doses tested, vegetal extracts had little effect on the expression of catabolic genes. Only curcumin slightly counteracted the effects of LPS on MMP-13 mRNA and protein release. However, the combination of curcumin with bromelain and harpagophytum reversed the effects of LPS on the mRNA levels of MMP-1, MMP-3 and MMP-13, and on the release of MMP-3 and MMP-13 proteins (Fig. 5). These data suggested that the combination of curcumin, bromelain and harpagophytum may reduce cartilage degradation during the OA process.

### The combination of curcumin with bromelain and harpagophytum significantly reduced the LPS-induced expression of genes associated with inflammation and pain

**Fig. 6:**
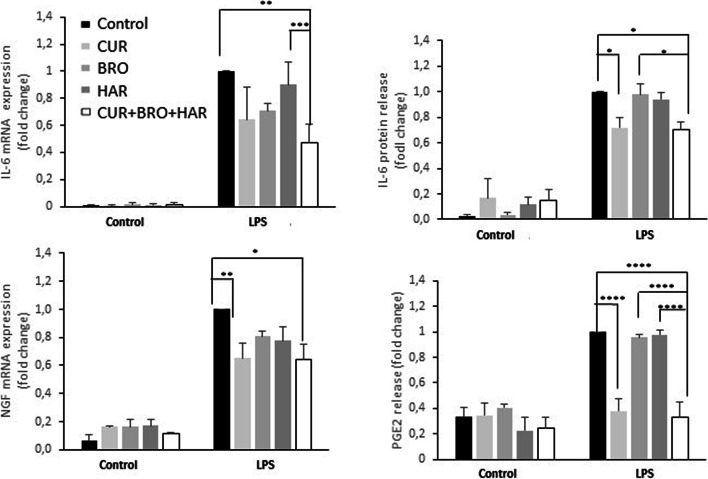
The combination of curcumin with bromelain and harpagophytum significantly reduced the LPS-induced expression of genes associated with inflammation and pain. Human synovial cells were treated with LPS (1 μg/ml) for 24 h in the presence of curcumin (CUR, 13 μM), bromelain (BRO, 14.7 μg/ml) and harpagophytum (HAR, 36 μg/ml), and all three together. At the end of the experiments, RNA were extracted and the media collected. Relative mRNA expression of NGF and IL-6 were determined by RT-PCR. Culture media were also collected and ELISA performed to assayed IL-6 and PGE2 release in medium. Values were compared to LPS-treated cells and presented as relative release (compared to the LPS group). *n* = 3. *: *p*-value< 0.05, **: *p*-value< 0.01, ***: *p*-value< 0.001

Next, we investigated the effect of these vegetal compounds on the expression of genes involved in inflammation and pain (Fig. 6). We observed that only curcumin was able to slightly reduce the LPS-induced expression of NGF and the release of PGE2 and IL-6. Interestingly, the combination of all three vegetal compounds (curcumin, bromelain and harpagophytum) significantly reduced the gene expression of IL-6 and NGF mRNA expression. It also decreased the IL-6 release and the production of PGE2. This suggests that the combination of these three compounds may reduce inflammation and pain.

## Discussion

To date, no efficient treatment exists to reverse osteoarthritis. As a result, it is crucial that we identify strategies that can slow down OA progression and that are usable in the long term. Some natural compounds are known to present anti-oxidative and anti-inflammatory actions, so they may be an alternative to pharmacological drugs. In this study, after proteomic characterisation of the in vitro OA model which was used, and after confirming that it was able to induce changes in gene expression profiles similar to that observed during OA, we demonstrated that the combination of curcumin, and bromelain and harpagophytum is efficient in counteracting numerous LPS-induced effects in human OA synovial cells.

Firstly, we evaluated the potential of lipopolysaccharide to induce changes in gene/protein expression by mimicking some features of OA. LPS is an endotoxin and a classical activator of the innate immune system. Because of its pathophysiological properties, LPS has been used to induce arthritis in conjunction with collagen in animal models [37, 38]. More recently, researchers have started to connect LPS with the pathogenesis of OA [39]. LPS is released by gut microbiota and is correlated with the pathophysiology of osteoarthritis, in part through the activation of macrophages. In addition, local LPS administration to joints induces synovitis and is used as a model to evaluate potential treatments for acute synovitis [40].^.^

Since LPS is now considered a trigger for OA pathology, especially by activating synovial cells, we have proposed that stimulated human OA synovial cells may induce inflammation and reproduce in vitro some changes observed during the OA process. Using proteomics, we demonstrated here that treating human OA synovial cells with LPS induces the expression of OA signature genes, and in particular reproduces some gene expression changes observed in synovial cells from inflammatory and normal areas of the osteoarthritis synovial membrane. A more targeted strategy showed, for instance, that LPS induced the expression of MMPs, IL-6, PGE2 and NGF, which are mainly markers of catabolism, inflammation and joint pain. Consequently, the stimulation of human OA synovial cells by LPS appeared to be a good in vitro model for studying inflammation during OA. With the knowledge that alleviating inflammation may prevent the onset or minimise the progression of OA [2, 14, 15, 39], we suggested the use of this in vitro model to test the ability of several natural substances to reduce inflammation.

Firstly, we demonstrated that curcumin has some anti-catabolic and anti-inflammatory effects in human OA synovial cells. This correlates with the literature, which demonstrates that curcumin reduces MMP-3 and MMP-13 expression in rabbit chondrocytes and in the articular cartilage of oestrogen-deficient rats, preventing collagen degradation [41, 42]. Also, curcumin prevents the activation of nuclear factor kappa B (NF-κB), the major mediator of inflammation [42, 43]. Another study shows that curcumin favours cartilage anabolism by increasing type II collagen synthesis [25, 44].

We also investigated the effects of *harpagophytum,* commonly known as devil’s claw, a plant used worldwide as a traditional remedy for joint pain associated with OA and mild rheumatic ailments [28, 45, 46]. Moreover, it has been described as having analgesic effects on neuropathic pain in rats [47]. We also studied the effects of bromelain, a food supplement that is sometimes described as an alternative treatment to nonsteroidal anti-inflammatory drugs (NSAIDs) [48]. Bromelain has analgesic properties [49, 50] and relieves OA symptoms [27]. However, at the dose tested, neither harpagophytum nor bromelain showed significant effects on the expression of studied genes, including NGF or PGE2, which are known to be related to joint pain. However, the combination of these vegetal components with curcumin may counteract numerous LPS effects in human OA-stimulated cells. The combination of curcumin with bromelain and harpagophytum significantly reduced the LPS-induced expression of genes associated with inflammation and pain, but also catabolism. This reinforced action of curcumin in combination with other natural compounds has already been seen [18]. For instance, the combination treatment of *Lactobacillus acidophilus* LA-1, vitamin B and curcumin ameliorates the progression of osteoarthritis by inhibiting the pro-inflammatory mediators [26]. However, to our knowledge, this paper is the first to show the benefits of combining curcumin with bromelain and harpagophytum.

In conclusion, we have described the changes in protein expression induced by LPS in human OA synovial cells and demonstrated that they are characteristic of inflamed OA synoviocytes, suggesting that this in vitro model may be useful for evaluating inflammation during OA. In addition, we have shown that the combination of three natural vegetal components reduced the expression of genes involved in catabolism, inflammation and pain, suggesting that together, they may present a beneficial effect on OA patients by alleviating OA pain and synovial inflammation and reducing cartilage degradation.

## Supplementary Information

**Additional file 1.**

## Data Availability

The datasets supporting the conclusions of this article are included within the article and its additional file.
